# Mapping the Key Technological and Functional Characteristics of Indigenous Lactic Acid Bacteria Isolated from Greek Traditional Dairy Products

**DOI:** 10.3390/microorganisms10020246

**Published:** 2022-01-23

**Authors:** Christina S. Kamarinou, Olga S. Papadopoulou, Agapi I. Doulgeraki, Chrysoula C. Tassou, Alex Galanis, Nikos G. Chorianopoulos, Anthoula A. Argyri

**Affiliations:** 1 Institute of Technology of Agricultural Products, Hellenic Agricultural Organization—DIMITRA, 14123 Lycovrissi, Greece; christinakamarinou7@gmail.com (C.S.K.); olga_papadopoulou@aua.gr (O.S.P.); adoulgeraki@aua.gr (A.I.D.); ctassou@nagref.gr (C.C.T.); 2 Department of Molecular Biology and Genetics, Democritus University of Thrace, 68100 Alexandroupolis, Greece; agalanis@mbg.duth.gr

**Keywords:** lactic acid bacteria, multi-functional cultures, probiotics, yogurt sensory analysis, enzymatic activity, antimicrobial activity

## Abstract

The aim of the current study was to isolate indigenous lactic acid bacteria (LAB) from traditional Greek cheeses and assess their biochemical, technological, and functional characteristics, so as to develop novel cultures with multi-functional properties. Hence, 109 LAB isolates were recovered from traditional fresh cheeses and were evaluated in vitro for their gas production; proteolytic, lipolytic, and haemolytic activity; exopolysaccharide production (EPS); enzymatic potential; and ability to grow at 6.5% NaCl and at different pH, temperature, and anaerobic conditions. Consequently, 48 selected isolates were further evaluated for their survival under simulated gastrointestinal tract conditions, partial bile salt hydrolase activity, antibiotic resistance, and antimicrobial activity against pathogens. These isolates were also incorporated as co-cultures in yogurt production to examine their sensory characteristics and their survival in the product. Some prominent isolates that showed favorable technological and functional characteristics (good survival rates at low pH and bile salts, ability to produce *β*-galactosidase, and EPS) and attributed desirable sensory characteristics to yogurt were *Lactococcus* *lactis* (SRX2, SRX3, SRX5, and SMX16), *Lactobacillus paracasei* SRX10, and *Lactiplantibacillus* *plantarum* (FRX7, FB1), while *Leuconostoc mesenteroides* FMX3 and *L. lactis* SMX2 showed an anti-listerial activity in vitro. The results of the present study are promising for the production of novel dairy functional products with an enhanced quality and safety.

## 1. Introduction

Fermentation is the oldest, most reliable, and inexpensive method of food processing, with the main goal being food preservation [1]. Today, there are over 3500 traditionally fermented foods with a wide variety of flavors, aromas, and textures, which makes them unique [2,3]. The industrialization of food fermentation began with the discovery of yeasts and bacteria as food fermenting organisms, back in 1857 by Louis Pasteur [4]. Nowadays, food fermentation is defined as a food processing technology in which the growth and metabolic activities of microorganisms are used to preserve food [5,6] and to improve the palatability of the food, the richness of the proteins and carbohydrates, and the bioavailability of vitamins and minerals [7,8,9]. Continuous studies on fermented foods have led to the conclusion that the health-promoting substances are associated with the microorganisms that contribute to the fermentation of these foods, as well as the substances they produce [10]. Βacteria that are widely used in the food fermentation industry are lactic acid bacteria (LAB) [11], and some of their characteristics are the competitive ability to have a low pH; the acid production (i.e., lactic acid); and the production of primary and secondary antimicrobial metabolites, such as bacteriocins, hydrogen peroxide, diacetyl, and CO_2_. All of the above can play a role in LAB competition with other microorganisms during fermentation [12].

Based on their aforementioned properties, LAB have been extensively studied and a variety of LAB genus and/or species are granted in the US with a “generally recognized as safe” (GRAS) or in the EU with a “qualified presumption of safety” (QPS) status. The GRAS status is most given to *Lactococcus* and *Lactobacillus*, while for other LAB genera like *Streptococcus*, certain species have been granted a GRAS/QPS status; however, none of the species of the genus *Enterococcus* have yet been granted a GRAS/QPS status [13], due to the probability of containing opportunistic pathogens [14].

Consumer awareness about fermented foods has increased because of their nutritional and health benefits, and the consumption of fermented foods worldwide is estimated to constitute one third of the human diet [15]. Utilizing LAB as bio-preservatives/protective cultures in food fermentations can be beneficial for improving the microbial safety of food by competing foodborne pathogens, as well as by favoring the sensory and nutritional characteristics of the implemented fermented foods [15]. In parallel, selected probiotic LAΒ strains with recognized beneficial health effects after human consumption (i.e., maintenance of balance of the host’s intestinal microbiota and prevention of infections caused by intestinal pathogens) may also be used by either adding them directly to foods as food additives [16], or, if suitable, as starter cultures in the manufacture of food. According to the Food and Agriculture Organization/World Health Organization, probiotics are “live microorganisms which when administered in adequate amounts confer a health benefit on the host” [17]. This definition was adopted by the International Scientific Association for Probiotics and Prebiotics (ISAPP) in 2013 [18]. Among the various types of microorganisms that are used as probiotics, selected strains of *Lactobacillus acidophilus*, *Lactobacillus sporogenes*, *Lactobacillus paracasei*, *Lactiplantibacillus plantarum*, *Lacticaseibacillus rhamnosus*, *Limosilactobacillus reuteri*, *Limosilactobacillus fermentum*, *Levilactobacillus brevis*, *Lacticaseibacillus casei*, *Lactococcus lactis* subsp. *cremoris*, and *Streptococcus salivarius* are used for the production of fermented dairy products [19,20]. The health benefits offered by LAB can be nutritional and/or therapeutic, including vitamin production, allergies, and immunoregulation [21]; relief of lactose intolerance symptoms [22]; reduction in the risk of Crohn’s disease [23] and diabetes [24]; or even anti-cancer properties [25].

As reported by several studies, dairy fermented food products are the most traditional source for the isolation of probiotic microorganisms [2,26]. However, several studies have also isolated potential probiotic bacteria from many other fermented products, such as table olives [27,28], fermented meat [29,30], vegetables and fruits [31,32], and fermented cereals [33,34], indicating that the isolation and screening of “wild” strains originating from a variety of food sources can be used as a powerful tool to obtain new strains (with multi-functional properties). This indigenous LAB microbiota can be applied as starters or as adjunct cultures with multi-functional properties (technological/protective/probiotic) in food fermentation by combining one or more strains.

Based on the above, the aim of this work was to isolate indigenous LAB from artisanal traditional Greek cheeses and to evaluate their biochemical, technological, and probiotic characteristics, so as to develop novel specific bacterial cultures for the production of dairy products with enhanced quality and distinctive organoleptic characteristics. For this reason, 109 isolates were recovered from two cheeses and were tested with a series of in vitro tests, while isolates that exhibited moderate or good properties regarding the in vitro tests were further investigated in situ (yogurt) to examine their sensorial characteristics and their presence in the product after production (enumeration of microbial population and estimation of the isolates recovery rate, using random amplified polymorphic DNA (RAPD) PCR).

## 2. Materials and Methods

### 2.1. Microbiological and pH Analyses from Traditional Artisanal Greek Cheeses

Traditional Greek cheeses, i.e., fresh (first ripening stage) white brined cheese from Crete and fresh (first ripening stage) semi hard goat cheese from Serifos, were obtained from artisanal producers. All cheese samples were produced following the traditional procedure without the use of commercial starter cultures. Samples were transported to the laboratory under refrigeration with minimal delay, and microbiological analyses and pH measurement were performed. In brief, 25 g of cheese samples were aseptically transferred to 225 mL quarter strength Ringer’s solution (LABM, Lancashire, UK) and homogenized in a stomacher device (Stomacher 400 circulator, Seward Limited, Norfolk, UK) for 60 s at room temperature. The resulting suspensions were serially diluted in the above diluent (Ringer’s solution) and 0.1 or 1 mL of the sample was spread or poured in triplicate on the following agar media: (i) Plate Count Agar (PCA, 4021452, Biolife, Milano, Italy) for Total Aerobic Viable Counts (TVC), incubated at 30 °C for 48–72 h; (ii) De Man-Rogosa and Sharpe Agar (MRS ISO agar, LabB223, LABM, Lancashire, UK) overlayed with the same medium and incubated at 30 °C and 42 °C for 48–72 h for the enumeration of mesophilic and thermophilic LAB rods, respectively; (iii) M17 Agar (M17, 4017192, Biolife, Milano, Italy) incubated at 37 °C and 42 °C for 48–72 h for the enumeration of mesophilic and thermophilic LAB cocci, respectively; (iv) Streptomycin Thallous Acetate-Actidione Agar (STAA, Biolife, Milano, Italy) for the enumeration of *Brochothrix thermosphacta*, incubated at 25 °C for 72 h; (v) Rose Bengal Chloramphenicol Agar (RBC Agar, Biokar diagnostics, Allonne, France) for the enumeration of yeasts and moulds, incubated at 25 °C for 72 h; (vi) Violet Red Bile Glucose Agar (VRBGA, Biolife, Milano, Italy) for *Enterobacteriaceae*, overlaid with the same medium and incubated at 37 °C for 18–24 h; (vii) Baird Parker Agar (BP, LABM, Lancashire, UK) with Egg Yolk Tellurite (X085, LABM, Lancashire, UK) for coagulase-positive staphylococci incubated at 37 °C for 48 h; (viii) Pseudomonas Agar Base with selective supplement (PAB, Biolife, Milano, Italy) for *Pseudomonas* sp., incubated at 25 °C for 48–72 h; (ix) Palcam Agar (Palcam Agar, Biokar Diagnostics, Allonne, France) with Palcam selective supplement (BS00408, Biokar Diagnostics, Allonne, France) for the enumeration of *Listeria monocytogenes* incubated at 37 °C for 24 and 48 h; and (x) Xylose Lysine Deoxycholate (XLD, Oxoid, Hampshire, UK) for *Salmonella* sp. incubated at 37 °C for 16–18 h. The cheese samples were also analyzed using enrichment methods for ensuring the absence of *Salmonella* sp. according to ISO 6579-1:2017 and *L. monocytogenes* according to ISO 11290-1:2017. Finally, the pH value was recorded after the end of microbiological analysis, with a digital pH meter (HI2211, pH/ORPMeter, HANNA instruments, Woonsocket, RI, USA) by immersing the glass electrode in the cheese homogenate. 

### 2.2. Isolation of Lactic Acid Bacteria from Traditional Artisanal Greek Cheeses

From each cheese sample examined, approximately 20% of the colonies from the appropriate dilution of MRS agar and M17 agar were selected and purified and then stored at −80 °C in MRS broth (MRS broth, Biokar diagnostics, Allonne, France) or M17 broth (M17 broth, Biokar diagnostics, Allonne, France) supplemented with 20% (*v*/*v*) glycerol (APPLICHEM, Darmstadt, Germany) [35]. A total of 109 isolates were recovered, in detail, 64 isolates from white brined cheese and 45 isolates from semi hard goat cheese.

### 2.3. Biochemical, Technological Characteristics, and Haemolytic Activity of Cheese Isolates

#### 2.3.1. Biochemical and Technological Characteristics

The 109 cheese isolates were screened for their biochemical/technological characteristics. All of the isolates were initially tested for colony morphology, Gram-stain morphology, and catalase and oxidase reactions. Additionally, a series of tests was performed including growth in MRS and M17 broths with pH adjusted values to 4.4 and 9.6 to discriminate the isolates belonging to the genus *Enterococcus* according to Giraffa [36], growth at 10 and 42 °C, anaerobic growth, growth in broths containing 6.5% NaCl, the ability of gas production [37], diacetyl production using citrate [38], proteolytic activity [38], lipolytic activity [38], and exopolysaccharides (EPS) production (technological and probiotic property) [39].

#### 2.3.2. Haemolytic Activity

The 109 cheese isolates were also evaluated for their haemolytic activity. Fresh bacterial cultures were streaked on Columbia agar plates containing 5% (*w*/*v*) horse blood (OXOID, Hampshire, UK) and incubated for 48 h at 30 °C. Blood agar plates were examined for signs of *β*-haemolysis (appeared as clear zones around colonies), *α*-haemolysis (green-hued zones around colonies), or *γ*-haemolysis (no zones around colonies). The test was performed in triplicate [40].

Isolates that were able to grow in broths with the pH adjusted to 9.6 (61 isolates), i.e., putative enterococci, were excluded from further analysis, so the current study continued with the remaining 48 isolates. All of the tests were performed in triplicate.

### 2.4. Identification and Characterization of the LAB Isolates with Molecular Tools

The selected 48 isolates and a representative percentage of isolates (10%) that grew on MRS and M17 broths with the pH adjusted to 9.6 were molecularly identified at the genus or species level. DNA was extracted according to Cocolin et al. [41]. Four microliters of the DNA extracted from the isolates were subjected to sequence analysis of the V1–V3 region of 16S rRNA gene, according to Doulgeraki et al. [42]. The sequences were analyzed using the online program BLAST to classify phylogenetically and to identify the closest known related species of the 16S rRNA gene, by comparing the above gene sequences with other homologies deposited in the NCBI databases. For the differentiation of *L. plantarum*, *L. pentosus*, and *L. paraplantarum* (*L. plantarum* group), a species-specific multiplex PCR assay targeting the *recA* gene was employed [43], where the expected amplicons were 318 bp for *L. plantarum*, 218 bp for *L. pentosus*, and 107 bp for *L. paraplantarum*. For the differentiation of *L.* *casei*, *L. rhamnosus*, and *L. paracasei* (*L. casei* group) a specific multiplex PCR assay targeting the *tuf* gene was employed [44], where the sizes of the expected amplicons were 700, 540, and 350 bp for *L. casei*; 540 and 240 bp for *L. paracasei;* and 540 bp for *L. rhamnosus*. Amplicons stained with GelRed (6X GelRed^®^ Prestain Loading Buffer with Tracking Dye, Biotium, California, USA) were separated after electrophoresis in agarose gels (2% *w*/*v*) in TAE 1X at 100 V for 1 h. DNA profiles were photographed using a Gel Doc System (Gel Doc Go Imaging System, Bio-Rad, Hercules, CA, USA).

### 2.5. Enzymatic Activity

The 48 selected isolates were examined for enzymatic activity using the API ZYM test (API ZYM 25,200, BioMerieux, Marcy ľ Etoile, France) following the manufacturer instructions. All tests were performed in triplicate.

### 2.6. Survival under Conditions Simulating the Human GI Tract

#### 2.6.1. Resistance to Low pH and to Bile Salts

To examine the resistance of the isolates at a low pH, fresh bacterial cells were suspended in a Phosphate Buffered Saline (PBS) buffer with pH adjusted to 2.5, as described before [40]. In brief, resistance was enumerated after incubation at 37 °C in MRS and M17 agar plate (depending on each microorganism) for 0 and 3 h, reflecting the time that food spends in the stomach. To examine the resistance to bile salts, fresh bacterial cells were suspended in a PBS buffer with pH adjusted to 8 containing 0.5% (*w*/*v*) bile salts (LP0055, OXOID, Hampshire, UK) consisting mainly of sodium glycocholate and sodium taurocholate [40]. Resistance was enumerated after incubation at 37 °C in MRS and M17 agar plates (depending on each microorganism) for 0 and 4 h, reflecting the time that food spends in the small intestine. All tests were performed in triplicate.

#### 2.6.2. Bile Salts Hydrolysis

Fresh bacterial cultures were streaked on MRS and M17 agar plates containing 0.5% taurodeoxycholic acid (TDCA) (T0875, Sigma-Aldrich, St. Louis, MO, USA). The different colony morphology (partial hydrolysis) revealed that the hydrolysis effect differentiated from the control MRS and M17 agar plates after 48 h of anaerobic incubation at 37 °C. The test was performed in triplicate.

### 2.7. Antibiotic Resistance

Τhe LAB isolates were inoculated (1%, *v*/*v*) in MRS and M17 broths supplemented with antibiotics (chloramphenicol, kanamycin, erythromycin, tetracycline, clindamycin, vancomycin, streptomycin, gentamicin, and ampicillin) at various concentrations (1, 2, 4, 8, 16, 32, 64, 128, 256, 512, and 1024 μg/mL) according to the European Food Safety Authority [45] and examined for growth in a microplate reader (SpectraMax Plus 384 Microplate Reader, Molecular Devices, Sunnyvale, VA, USA) (Optical Density-OD at 610 nm) following a 24 h incubation period at 30 °C or 37 °C (depending on the optimum temperature growth of each microorganism). The resistance of each microorganism in the presence of antibiotics was noted by the absorbance measurements that were recorded after 24 h of incubation. Wells containing only MRS or M17 with or without the antibiotics were used as the negative controls, while wells inoculated with each LAB isolated without the addition of antibiotics served as the positive controls. All tests were performed in triplicate.

### 2.8. Antimicrobial Activity against Pathogens

To examine the antimicrobial activity of the 48 LAB isolates, the agar well diffusion assay was performed according to Pavli et al. [40]. An initial inoculum of 6 log CFU/mL of the target foodborne pathogens was incorporated into BHI soft agar (1%) plates. Then, 50 μL of (a) fresh bacterial cells, (b) cell free supernatants (CFS), and (c) CFS with pH adjusted to 6.5 of the LAB isolates were transferred in 5 mm diameter holes drilled into the agar. The dishes were incubated at 37 °C for 24 h and the antimicrobial activity was observed by the inhibition zones around the well. As a positive control, the antibiotic kanamycin (30 μg/mL) was used, while MRS and M17 broths adjusted to pH 6.5 were the negative controls. The foodborne pathogens that were studied were *Salmonella enterica* subsp. *enterica* serovar Enteritidis FMCC-B56, *Staphylococcus aureus* FMCC-B202 and *Listeria monocytogenes* FMCC-B129, FMCC-B131, and FMCC-B133 (kindly provided by the laboratory of Food Microbiology and Biotechnology of the Agricultural University of Athens), and DSMZ19094 and DSMZ15675 (German Collection of Microorganisms and Cell Cultures, Braunschweig, Germany). All tests were performed in triplicate.

### 2.9. Yogurt Fermentation Trials Using Selected LAB Isolates

#### 2.9.1. Preparation of Yogurt

Yogurts were prepared by using pasteurized and homogenized bovine milk that was additionally heated at 80 °C for 30 min, rapidly cooled to 45 °C, and inoculated with the commercial yogurt culture (*Str. thermophilus* and *L. bulgaricus*) and with the addition of the 48 LAB isolates per case, according to Saxami et al. [46]. In brief, all LAB cultures were revived from a stock culture stored at −80 °C, into 10 mL of MRS or M17 broths and incubated overnight at 30 °C and 37 °C, respectively. The subcultures were prepared in fresh 10 mL MRS or M17 broths and incubated for 24 h at 30 °C and 37 °C, respectively. For milk inoculation, the cells were harvested by centrifugation (6000× *g*, 5 min, 4 °C), washed twice with ¼ strength Ringer’s solution and resuspended in milk to give a final population of approximately 6 log CFU/mL for the production of the control (commercial yogurt culture) and the 48 yogurt trials. The corresponding samples were incubated in appropriate conditions (42 °C, 6 h, pH 4.6) and after the fermentation process the yogurt samples were stored at 4 °C for 24 h and consequently subjected to sensory analysis. The samples with acceptable sensory properties (≥5 point in hedonic scale) (19 cases and the control case) were subsequently subjected to microbiological (LAB, thermophilic LAB cocci) and molecular analyses. The experiment was performed twice (two different milk batches) with three sample replicates per case.

#### 2.9.2. Sensory Analysis

The organoleptic evaluation of the yogurt samples was performed by a group of seven people (staff of the laboratory) who were previously trained in evaluating dairy products [47]. The organoleptic assessment of the yogurts was performed after 24 h of yogurt production, under artificial light in individual booths in a special sensory analysis room allocated at the Institute of Technology of Agricultural Products. Yogurts were served in plastic cups, codified with two random digits. Panelists used unsalted crackers and water to clean their palates between the samples. The overall perception of appearance, aroma, taste, and texture of the yogurts, as well as specific indicators of each sensorial attribute, were assessed from the panel using a 10-cm hedonic scale. In detail, the evaluation of specific indicators; appearance, i.e., white color, skin, and syneresis (serum release); texture, i.e., grainy, consistency, and homogeneity; aroma, i.e., buttery, acidic and animal aroma, and acid; and taste, i.e., sweet, bitter, salty, and rancid. The samples were scored in the 10-cm intensity scale, where the direction of the hedonic scale was from left to right with increasing intensities, i.e., weak to strong, little to much, etc. Scores regarding the overall perception of the appearance, aroma, taste, and texture of the yogurts were also recorded. Samples were considered acceptable when their total scores in the overall perception of the appearance, aroma, taste, and texture of the yogurts were above 5.

#### 2.9.3. Isolation of LAB and Molecular Analysis

A total of 223 LAΒ isolates were randomly collected from the highest countable dilution of MRS or M17 agar plates of the yogurt samples (19 cases) with sensory scores (overall perception) above five in the hedonic scale. All the isolates were stored at −80 °C in MRS or M17 broth supplemented with 20% (*v*/*v*) glycerol, until further use. One hundred nanograms of the DNA extracted from the isolates were subjected to RAPD-PCR analysis using the primer M13 (5′-GAGGGTGGCGGTTCT-3), according to Giraffa et al. [48]. Amplicons stained with GelRed (6X GelRed^®^ Prestain Loading Buffer with Tracking Dye, Biotium, CA, USA) were separated after electrophoresis in agarose gels (2% *w*/*v*) in TAE 1X at 120 V for 2 h. A 1 kbp plus DNA Ladder (New England Biolabs, Ipswich, MA, USA) was used as a DNA molecular weight marker. DNA profiles were photographed using a Gel Doc System (Gel Doc Go Imaging System, Bio-Rad, Hercules, CA, USA). Profiles of the 19 LAB isolates and profiles of the commercial yogurt culture were used as the references to compare the profiles of the LAB isolates recovered from yogurt samples.

## 3. Results and Discussion

### 3.1. Cheese Microbiota of the Traditional Artisanal Greek Cheeses

The dominant population of the indigenous microbiota in the cheese samples consisted of mesophilic LAB rods (8.5 and 8.4 log CFU/g in white brined cheese and in goat cheese, respectively) and LAB cocci (7.8 and 8.4 log CFU/g in white brined cheese and in goat cheese, respectively), thermophilic LAB rods (6.3 and 7.4 log CFU/g in white brined cheese and goat cheese, respectively), and thermophilic LAB cocci (5.8 and 7.6 log CFU/g in white brined cheese and in goat cheese, respectively). The population of the remaining examined microbiota consisted of yeasts and molds with a population level of 5.1 and 5.6 log CFU/g, and staphylococci with populations of 5.0 and 6.1 log CFU/g, for white brined cheese and goat cheese, respectively. It has to be noted that *Brochothrix thermosphacta* and *Pseudomonas* spp. were below the detection level of the enumeration method (<2.0 log CFU/g) for both cheeses. Additionally, *Enterobacteriaceae* were detected only in white brined cheese in a population of 3.1 log CFU/g. With regards to the examined pathogens, neither *Salmonella* spp. nor *L. monocytogenes* were detected in any of the samples after enrichment. Finally, the pH values were 5.39 and 5.16 in the white brined cheese and in goat cheese, respectively.

In the microbial ecology of cheese, the microbiota provides important knowledge about the quality of raw milk, the interference of production technology, the equipment used, and the environmental conditions [49]. LAB usually predominate among other microbes [50] and this coincides with the aforementioned results. The presence of yeasts and molds is expected in cheese at the first stage of ripening, when the microbial slime starts appearing [51]. The presence of staphylococci and *Enterobacteriaceae* can be due to many factors, such as thermal treatments, the water activity, and pH, as well as the general condition of the manufacturing facility [50]. It is worth noting that after a month of ripening, bacteria such as *Enterobacteriaceae*, coliforms, staphylococci, and yeasts decrease their numbers rapidly due to the low pH and high salt [51]. Major pathogenic bacteria like *Salmonella* spp. and *L. monocytogenes* were not detected, which means that the two artisanal cheeses are considered as safe, according to the European Union regulatory criteria (EC 1141/2007).

Similar results with the present study were found previously [47,52]. For instance, Papadopoulou et al. [47] observed that during first ripening period of feta cheese, mesophilic LAB were found to be above 7.0 log CFU/g, yeasts and molds were 4.0 log CFU/g, and the staphylococci population was 6.5 log CFU/g. Similar results were observed for the traditional Greek cheeses named “Kalathaki” and “Melichloro” during first week of ripening, where mesophilic LAB cocci were found in a higher population than thermophilic LAB cocci, mesophilic LAB rods were in higher population than the thermophilic LAB rods, yeasts and molds population was ca. 3.0–4.0 log CFU/g, and *Staphylococcus* spp. was estimated ca. 6.4–6.8 log CFU/g [52].

### 3.2. Biochemical, Technological, and Molecular Characterization of LAB Isolates

A total of 109 isolates were recovered from the two cheeses (64 isolates from white brined cheese and 45 isolates from goat cheese) and a set of biochemical and technological tests were performed (Supplementary Tables S1 and S2). All of the tested isolates were found to be Gram-positive, catalase-negative, and oxidase-negative. Morphologically, the isolates were coccobacilli (53.2%), cocci (36.7%), and rods (10.1%), and the cells were arranged either single or in pairs (coccobacilli, cocci, and rods), in tetrads (coccobacilli and cocci), or in chain forms (rods). It was observed that all the isolates were able to grow under anaerobic conditions and in MRS or M17 broths with adjusted to pH of 4.4. Additionally, the majority of isolates grew at 10 °C (99 isolates), at 42 °C (71 isolates), in the presence of 6.5% NaCl (81 isolates) and showed proteolytic activity (65 isolates). None of the isolates were able to grow on Simmons’ Citrate Agar (diacetyl production) and also no lipolytic activity was observed. Furthermore, 19 isolates produced exopolysaccharides (EPS) and 33 isolates produced gas from glucose. Moreover, 61 isolates were able to grow in MRS or M17 broths with adjusted pH to 9.6 (presumptive enterococci). Enterococci can be distinguished from streptococci, lactococci, lactobacilli, and leuconostocs by their ability to grow at an alkaline pH (9.6) [53]. Enterococci comprise one of the main genera of the LAB group and are ubiquitous in many fermented foods, especially in artisanal dairy products [38,54]. They are also involved in the development of organoleptic characteristics of traditionally manufactured cheeses and dry sausages and, nowadays, most of them are used as probiotics [55]. At the same time, recent reviews reported that the use of enterococci in the food industry has raised doubts about their safe application, since they have been associated with several human infections [55,56]. Still, the industrial application of enterococci as starter or adjunct cultures, as well as of probiotics, for manufacturing dairy products is an issue that must be carefully addressed by the industry [36,57]. Factors that appear to be responsible for enterococcal pathogenesis are involved in adhesion, translocation, and immune evasion [58]. To date, this genus has not yet obtained “GRAS” status [57]. As a result, in this research, it was considered necessary to exclude these microorganisms from further analysis, as well as not to use them as co-starters/adjunct cultures in the production of yogurt. Consequently, from the 109 isolates, 48 isolates were selected for further investigation and 61 presumptive enterococci isolates were excluded.

The remaining 48 isolates, as well as 6 representative isolates of presumptive enterococci (due to their ability to grow on pH 9.6) were identified by sequencing the V1–V3 region of the 16S rRNA gene. The sequence analysis of the 16S rRNA gene of the 48 isolates revealed the presence of *L. mesenteroides* (*n* = 25), *L. pseudomesenteroides* (*n* = 4), *Leuconostoc* sp. (*n* = 1), *L. plantarum* group (*n* = 5), *Levilactobacillus brevis* (*n* = 2), *L. casei* group (*n* = 1), and *L. lactis* (*n* = 10) (Supplementary Tables S1 and S2). For the representative six isolates with the ability to grow at pH 9.6, the sequence analysis of the 16S rRNA gene confirmed that they belonged to the genus *Enterococcus* spp. (Supplementary Tables S1 and S2). According to the multiplex PCR assay targeting the *recA* gene (differentiation of *L. plantarum* group), all the isolates (five) were characterized as *L. plantarum* (Supplementary Figure S1A). For the differentiation of the sole isolate that was assigned to the *L. casei* group, the multiplex PCR assay targeting to the *tuf* gene showed that the isolate was identified as *L. paracasei* (Supplementary Figure S1B).

According to previous studies dealing with the identification of the microbiota of dairy artisanal products, the most frequently isolated LAB were *L. lactis* (44%), *L. mesenteroides* (23%), *Lactobacillus* spp. (23%), and *Enterococcus* spp. (10%) [51]. Comparable results with the present study were obtained by other authors, which showed that in the white brined cheeses, regardless of the ripening period, the most common LAB genera were identified as *Lactobacillus*, *Lactococcus*, *Enterococcus*, and *Leuconostoc* [59,60]. Furthermore, in another study concerning the microbiota of fresh feta cheese, identification of the LAB isolates included *L. lactis* (77.6%), *E. faecalis* (15.5%), and *Lactococcus garvieae* (3.5%) [61]. Additionally, in a study dealing with the characterization of the microbiota of feta cheese during ripening and storage, *Enterococcus* spp. were found in high numbers during the first ripening stage and their population declined during the second ripening stage, however their presence was still evident in low population levels in the mature cheese [62]. Moreover, it was observed that enterococci as ubiquitous LAB can occur frequently in large numbers in dairy products [51,63], and their presence is considered as an indication of insufficient sanitary conditions during the production and processing of milk [38].

On the other hand, in another study concerning fresh semi hard cheese (i.e., Kasseri), it was observed that only a percentage of 25% of the predominant LAB microbiota was identified as pediococci and lactobacilli, while 50% of the identified microbiota was found to be enteroccoci [51]. Moreover, Domingos-Lopes et al. [64] identified 114 isolates at the genus or species level that were retrieved from an artisanal semi hard cheese (Pico cheese from Portugal) during the early stage of ripening, and showed that the highest percentage of the examined isolates belonged to the *Enterococcus* genus (73.7%); 19.3% were identified as *L. paracasei* subsp. *paracasei*, *L. plantarum*, *L. paraplantarum*, and *L. otakiensis*; 4.4% were *L. mesenteroides* and *L. citreum;* and 2.6% were assigned to the *Lactococcus* genus.

Among the biochemical and technological characteristics of the 48 isolates, all of them were able to grow under anaerobic conditions, at pH 4.4 and at 10 °C, while 10 isolates belonging to *L. mesenteroides*, *L. plantarum*, and *L. brevis* were able to grow at 42 °C. Isolates belonging to *L. mesenteroides* (15 isolates) and *L. plantarum* (5 isolates) grew in the presence of 6.5% NaCl and isolates of *L. mesenteroides* (15 isolates), *L. plantarum* (2 isolates), and *L. lactis* (6 isolates) showed proteolytic activity. EPS production was observed in 6 isolates belonging to *L. pseudomesenteroides* (SRX1) and *L. lactis* (SRX2, SRX3, SRX5, SMX16, and SMX20). Finally, 28 isolates belonging to *L. mesenteroides*, *L. pseudomesenteroides*, *Leuconostoc* sp., and *L. brevis* produced gas from glucose (Supplementary Tables S1 and S2).

Technological characteristics, such as the catabolism of sugar and citrate, and the proteolytic and lipolytic activity are important traits for adding texture and aroma in fermented foods [64,65]. The results of the present study are related to previous studies dealing with LAB isolated from naturally fermented dairy products, where none of the LAB was found to catabolize citrate [66] and the majority of the LAB did not show a lipolytic activity [67]. Domingos-Lopes et al. [64] observed that enterococci, which were isolated from an artisanal semi hard cheese, showed lipolytic activity. Furthermore, in many studies dealing with the microbiota of semi hard and hard cheeses, *L. mesenteroides*, *L. lactis*, and *L. paracasei* exhibited proteolytic activity, a result that was also observed in the current study, while *L. plantarum* did not show proteolytic activity, on the contrary to the findings of the current research [64,68]. In respect to EPS production, various researchers observed that microorganisms belonging to the genera *Streptococcus*, *Lactobacillus*, *Lactococcus*, and *Leuconostoc* produced EPS [69,70]. In addition to these findings, Breyer et al. [68] noted that isolates of *L. mesenteroides* and *L. lactis* produced EPS, a result observed in the current study. LAB isolates that can produce EPS are receiving increasing attention from the industry due to their ability to improve the appearance, the stability, and the rheological properties in several fermented foods [71]. Furthermore, EPS are the major components of the bacterial biofilm, which enhances the colonization of probiotic bacteria in the gastrointestinal tract, allowing the cells to express their probiotic abilities, and have also been reported to show antibiofilm activity against pathogens. For instance, Kim et al. [72] found that the EPS of *L. acidophilus* had a stronger antibiofilm activity against the growth of *E. coli*, *S.* Enteritidis, *S. typhimurium*, *Yersinia enterocolitica*, *Pseudomonas aeruginosa*, *L. monocytogenes*, and *Bacillus cereus*. Moreover, it was observed previously that LAB isolated from fermented foods, such as *Leuconostoc* spp. and *L. brevis*, produced gas during the degradation of glucose, while *L. plantarum* and *L. lactis* did not [73,74], which is in line with the findings of this research.

### 3.3. Haemolytic Activity

None of the examined 109 isolates exhibited *β*-haemolytic activity when grown in Columbia horse blood agar. Most of the isolates (98 isolates) were *γ*-haemolytic (i.e., no haemolysis), while 11 isolates did not grow on this substrate (Supplementary Tables S1 and S2). These results are in accordance with previous studies, where no haemolytic activity was observed from LAB isolates of a dairy origin [75]. The absence of haemolytic activity is considered as a necessary condition to assess the safety of potential probiotic isolates (FAO, 2006). Haemolytic activity is a common virulence characteristic among pathogens and it might break down the epithelial layer of the intestine, so exclusion of the isolates presenting *γ*-haemolysis is deemed necessary [76].

### 3.4. Enzymatic Activity

**Figure 1:**
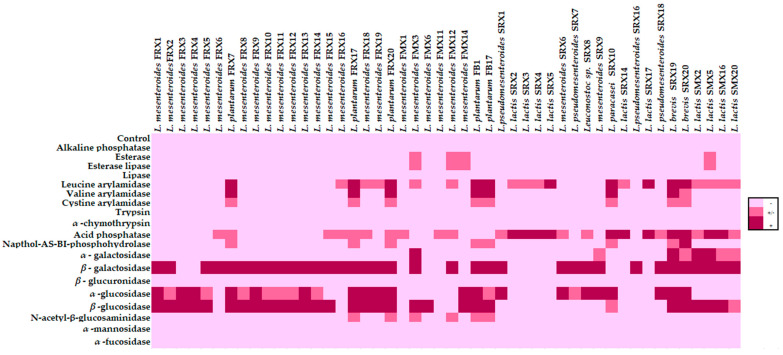
Enzymatic activity of the 48 LAB isolates determined by API ZYM 25,200. The color intensity of enzymatic reactions represents the enzymatic activity of each isolate: dark pink: “+” strong activity; pink: “+/-“ moderate activity; light pink: “-“ no activity.

In accordance with the above, the 48 selected isolates (isolates excluding enterococci) were further characterized for their enzymatic activity using the API ZYM 25,200 (Biomerieux), and the results are presented in Figure 1. It was evident that no enzymatic activity of the enzyme’s alkaline phosphatase, lipase, trypsine, *α*-chymothrypsin, *β*-glucurosidase, *α*-mannosidase, and *α*-frucosidase was observed. On the other hand, the enzymatic activity of leucine arylamidase, acid phosphatase, α-galactosidase, and *β*-galactosidase was detected in many isolates of the species *L. mesenteroides*, *L. plantarum*, *L. lactis*, *L. paracasei*, and *L. brevis.* For the rest of the enzymes (valine arylamidase, cystine arylamidase, napthol-AS-BI-phosphohydrolase, *N*-acetyl-*β*-glucosaminidase, esterase, and esterase lipase), only few microorganisms exhibited an enzymatic activity. More specifically, the activity of valine arylamidase, cystine arylamidase, and napthol-AS-BI-phosphohydrolase was noted only in *L. plantarum*, *L. paracasei*, and *L. brevis* isolates. The activity of *N*-acetyl-*β*-glucosaminidase was detected in all *L. plantarum* isolates and in three *L. mesenteroides* isolates, i.e., FMX3, FMX12, and FMX14, while the activity of esterase and esterase lipase was observed only in three isolates assigned to *L. mesenteroides*, i.e., FMX3, FMX12, and FMX14, and in *L. lactis* SMX5. It is also worth noting that all of the isolates of the species *L. plantarum*, *L. mesenteroides*, and *L. brevis* displayed an enzymatic activity in many of the examined enzymes.

Similar results were noted by Ryu and Chang [77], who observed that no enzymatic activity was evident by LAB isolated from a fermented vegetable (kimchi) of the enzymes alkaline phosphatase, *α*-chymotrypsin, *β*-glucuronidase, or *α*-fucosidase. In the same research, the enzymatic activity of the enzymes *N*-acetyl-*β*-glucosaminidase and *β*-galactosidase was also observed from *L. plantarum* isolates. Herreros et al. [78] reported that *L. plantarum*, *L. lactis* subsp. *Lactis*, and *L. casei* subsp. *casei* exhibited enzymatic activity of leucine arylamidase; *L. casei* subsp. *casei* and one strain of *L. lactis* subsp. *cremoris* showed enzymatic activity of valine arylamidase and *L. mesenteroides; L. lactis* exhibited enzymatic activity of esterase and esterase lipase; *L. lactis* subsp. *lactis* presented enzymatic activity of acid phosphatase; and finally, *L. plantarum* and *L. lactis* subsp. *lactis* showed enzymatic activity of *β*-galactosidase. Lipinska-Zubrycka et al. [79] found that the isolates of *L. brevis* and *L. casei* showed enzymatic activity of esterase, esterase-lipase, leucine arylamidase, valine arylamidase, acid phosphatase, napthol-AS-BI-phosphohydrolase, *β*- galactosidase, and *α*-glucosidase. It is worth noting that the production of some enzymes is associated with both negative and positive results in human health, as well as in the technological characteristics of the food. For example, *β*-glucuronidase and *β*-glucosidase activity may have negative effects in the colon by converting aromatic hydrocarbons and amines in active carcinogens, thus allowing for the development of colon cancer [80,81]. In contrast, *β*-galactosidase, which is released by probiotics, contributes to the relief of lactose maldigestion symptoms, as *β*-galactosidase hydrolyzes lactose to glucose and galactose [82,83]. Regarding the correlation between the technological properties and enzyme activity, Williams and Banks [84] observed that many isolates of non-starter lactic acid bacteria (NSLAB), previously isolated from cheddar cheese, showed a *β*-galactosidase activity. The presence of *β*-galactosidase released the attached sugars of the glycosylated k-casein and as a result, the released sugars could be used by the microorganisms as an energy source [84]. In addition, esterase and lipase activity may affect the technological characteristics (flavor) of the food, as the presence of these enzymes could increase the concentration of free fatty acids in cheeses, which affects cheese flavor [85,86].

### 3.5. Survival under Conditions Simulating the Human GI Tract

The initial population at time 0 (h) of all 48 isolates was ≥7.9 log CFU/mL (Table 1). After 3 h of exposure to pH 2.5, a great variability of isolate resistance was shown based on the enumerated final population (Table 1). In detail, six isolates showed high resistance with survival up to 72%, i.e., *L. mesenteroides* SRX6; *L. paracasei* SRX10; and *L. plantarum* FRX7, FRX17, FRX20, and FB1. A set of 27 isolates assigned to *L. mesenteroides*, *L. pseudomesenteroides*, *L. lactis*, and *L. plantarum* showed moderate resistance with a survival range from 45 to 67%. The survival of the remaining 14 isolates assigned to *L. mesenteroides* and *L. pseudomesenteroides* species was less than 45%. Regarding resistance to bile salts, the results showed that the majority of the isolates (46) presented a survival rate over 72%, after 4 h of exposure to 0.5% bile salts (*w*/*v*) (Table 1). The remaining two isolates exhibited a survival rate of 63% (Table 1). Regarding bile salt hydrolase activity, 16 isolates out of 48 demonstrated partial bile salt hydrolase activity (Table 1). These isolates were *L. plantarum* FRX7, FRX17, FRX20, FB1, and FB17; *L. lactis* SRX2, SRX3, SRX4, SRX5, SMX2, SMX5, SMX16, and SMX20; *L. paracasei* SRX10; and *L. brevis* SRX19 and SRX20. The rest of the tested isolates did not exhibit bile salt hydrolase activity in the presence of 0.5% (*w*/*v*) taurodeoxycholic acid (Table 1).

The human stomach contains low pH values and these values can range from 1.5 to 4.5 during fasting or after a meal, respectively [87]. The potential probiotic bacteria, in order to exert their health benefits to the hosts, must be resistant to this acidic environment, with the first challenge being the resistance in low pH before reaching the intestinal tract [88]. The results of the current study are in agreement with previous studies, where *L. plantarum* and *L. paracasei* showed a very high resistance to low pH, compared to *L. mesenteroides* and *L. lactis*, which showed a lower resistance. Pavli et al. [40] observed that *L. plantarum* and *L. lactis* isolates showed higher resistance to low pH rather than the isolates of *L. mesenteroides*, where their final counts indicated the lowest resistance (3.0 log CFU/mL). Argyri et al. [27] also observed that most of *L. mesenteroides* and *L. pseudomesenteroides* isolates were found to be susceptible to a low pH, presenting population levels below 1.0 log CFU/mL after 3 h of exposure to pH 2.5. On the other hand, isolates of *L. plantarum*, *L. paracasei* subsp. *paracasei* with initial populations exceeding 8.0 log CFU/mL, were found to be resistant after 3 h of exposure to pH 2.5, exhibiting final populations of 6.0–8.0 log CFU/mL. In another study, LAB tolerance to acid stress varied between species and was caused by different protection mechanisms [89]. In the same study, it was observed that the presence of F0F1-AT-Pase, especially in the genus *Lactobacillus*, could make this genus more resistant than other LAB genus in acidic environments [89]. Similarly, other studies dealing with the resistance of LAB to bile salts found that the majority were resistant to 0.5% bile salts [27,40]. Moreover, Zoumpopoulou et al. [52] observed that all of the tested LAB isolates survived well in the presence of 1% (*w*/*v*) bile salts, showing less than 1.3 log CFU/mL reduction after 3 h of incubation at pH 8.0. The observed resistance in the presence of bile salts is of importance for the survival ability of the potential probiotic bacteria in the intestinal tract, as bile salts are present in the bile juice secreted by the gallbladder and end up in the duodenum [90]. The results regarding bile salt hydrolase activity are related with other studies, which found that *L. plantarum* and *L. lactis* strains isolated from dairy products showed a potential hydrolysis activity with sodium taurodeoxycholate [40,91]. Hydrolysis activity is a good indication for probiotics’ selection, as it can reduce cholesterol accumulation in humans [91].

### 3.6. Antibiotic Resistance

One of the main concerns regarding the use and safety of LAB isolates as probiotics in food production is their resistance to antibiotics [92]. In the case that bacteria are found to be resistant to several antibiotics, they pose a risk for horizontal transfer of the antibiotic resistance genes to pathogenic bacteria [93]. According to the breakpoints set by EFSA [45], the isolates are considered susceptible when they can grow at a concentration of a specific antimicrobial equal to or lower than the established cut-off value and are resistant when they are able to grow at a concentration of a specific antimicrobial higher than the established cut-off value [45]. In this study, the minimum inhibitory concentrations (MICs) of the 48 tested LAB isolates concerning antibiotic resistance are presented at Table 1. The results show that all isolates were susceptible to ampicillin and clindamycin, while for vancomycin, all LAB strains showed resistance, without a specified breakpoint from EFSA. However, all isolates belonging to *L. plantarum* showed susceptibility to streptomycin and resistance to gentamycin, kanamycin, erythromycin, and tetracycline, while all isolates except the isolate FB17 showed resistance to chloramphenicol. In addition, *L. brevis* SRX19 and SRX20 and *L. paracasei* SRX10 showed resistance to gentamycin, kanamycin, erythromycin, tetracycline, streptomycin, and chloramphenicol. In general, there may be differences between species and strains in resistance for several antibiotics. For instance, regarding *L. lactis* isolates, most of the isolates were resistant to gentamycin and erythromycin, while all of the isolates were found to be susceptible to tetracycline and chloramphenicol and resistant to streptomycin. All *L. lactis* isolates, except SRX2 and SRX3, were found to be resistant to kanamycin. Moreover, all *L. mesenteroides* isolates were found to be resistant to kanamycin; susceptible to tetracycline and chloramphenicol; and the majority of them susceptible to gentamycin, streptomycin, and erythromycin. In addition, all *L. pseudomesenteroides* isolates were resistant to kanamycin and susceptible to tetracycline and chloramphenicol, while isolates SRX1 and SRX7 were resistant to gentamycin and streptomycin, and SRX1 was resistant to erythromycin.

The results of the current study concur with past findings, where *L. plantarum*, *L. brevis*, *L. paracasei* and *L. lactis* showed resistance to gentamycin, kanamycin, tetracycline, and streptomycin [83,94]. On the other hand, the results of Pavli et al. [40] showed that *L. lactis* was found to be resistant only in vancomycin, whereas isolates of *L. plantarum* were found to be resistant to gentamycin, kanamycin, and tetracycline, and susceptible to streptomycin. In the study of Zoumpopoulou et al. [52], isolates of *L. mesenteroides* exhibited similar results with the current work, regarding the resistance and the susceptibility of these isolates in the examined antibiotics.

The emergence of probiotic strains resistant to antibiotics does not pose a threat to food or consumer safety, in fact their resistance to specific antibiotics that are, for instance, involved in antibiotic-induced diarrhea, may help to restore or maintain normal intestinal microflora [95,96]. In fact, antibiotic resistance may be a desirable property for strain selection, provided that the resistance genes are not transferable to other bacteria (especially pathogenic). According to previous studies, the antibiotic resistance observed for *Lactobacillus* strains are considered to be intrinsic or natural resistance, because they are chromosomally encoded and, therefore, they are not transmitted through horizontal gene transfer [95,96,97]. However, some probiotic species have yet to be studied [83]. The use of molecular techniques such as PCR and microarray analysis can be applied to determine the genotype of antibiotic resistance determinants and to classify the isolates as native or acquired [96]. The unravelling of the resistance mechanisms is a key to resolve these crucial issues in the future and to ensure their potential applicability in food systems.

### 3.7. Antimicrobial Activity against Pathogens

In this study, none of the fresh bacterial cells and the supernatants (CFS and CFS with pH adjusted to 6.5) of the 48 isolates showed an antimicrobial activity against the pathogens *Salmonella* Enteritidis (FMCC-B-56) and *Staphylococcus aureus* (FMCC-B-202). In contrast, two LAB isolates, i.e., *L. mesenteroides* FMX3 and *L. lactis* SMX2, inhibited the growth of all five *Listeria monocytogenes* strains (FMCC-B-129, FMCC-B-131, FMCC-B-133, DSMZ19094, and DSMZ15675). It was observed that both free cells and CFS exhibited an inhibition zone to the aforenoted strains of *L. monocytogenes*. It is known that many LAB have the ability to produce antimicrobial compounds, such as organic acids (lactic acid, acetic acid, formic acid, etc.), bacteriocins, sugar catabolites (ethanol, diacetyl, and carbon dioxide), and oxygen catabolites (hydrogen peroxide) [98]. The result that CFS with pH adjusted to 6.5 showed an antimicrobial action leads to the assumption that the presence of organic acids was not responsible for this antimicrobial activity. Future studies will involve the study of the mechanism responsible for the antimicrobial activity of the aforenoted LAB isolates.

Due to their antimicrobial ability, LAB can play a significant role in food preservation and in food safety improvement [59]. Breyer et al. [68] reported that one strain of *L. lactis*, isolated from dairy products showed an antagonistic activity against *L. monocytogenes* in vitro. Margalho et al. [99] observed that a *L. lactis* strain, previously isolated from artisanal Brazilian cheeses, exhibited an antagonistic activity in vitro against two different *L. monocytogenes* strains. In another study, the supernatants from two *Leuconostoc* bioprotective strains, previously isolated from fresh fruits and vegetables, showed in vitro and in situ (in Golden Delicious apple and Iceberg lettuce leaves) anti-listerial activity [100]. Finally, Sip et al. [101] reported that strains of *L. lactis* subsp. *cremoris*, *L. lactis* subsp. *lactis*, *L. garvieae*, *L. mesenteroides*, and *L. plantarum*, which were previously isolated from a traditional Polish cheese, displayed the highest anti-listerial activity in comparison with the other LAB isolates.

### 3.8. Yogurt Fermentation Trials Using Selected LAB Isolates

#### 3.8.1. Sensorial Characteristics of Yogurt Produced with the 48 Selected LAB

The 48 LAB isolates were included in yogurt fermentations as co-cultures to make an initial screening of their contribution to the organoleptic characteristics of yogurt, serving as a “model food”. In brief, after yogurt production, the samples were organoleptically evaluated by a seven-member panel and the results are presented in Table 2. Τhe yogurts were evaluated for their characteristics in terms of overall appearance, aroma, taste, and texture (Table 2), as well as for specific attributes, i.e., white color, skin, syneresis, buttery, acidic and animal aroma, acid, sweet, bitter, and salty and rancid taste, as well as grainy texture, homogeneity, and consistency (data not shown). Regarding the specific sensorial attributes for appearance, the results showed that all the samples exhibited differences in the attribute “syneresis”. Seven samples fermented with *L. mesenteroides* FRX15, FMX1, and FMX11; *L. pseudomesenteroides* SRX16; *L. plantarum* FRX17 and FB17; and *L. lactis* SRX14 were evaluated negatively due to this characteristic. The scores of the overall perception of appearance in the samples varied between cases. In detail, 11 samples were evaluated with the highest scores (>7) and the majority of these samples (6 out of 11) were produced with *L. lactis* SRX3, SRX4, SRX5, SRX17, SMX5, and SMX16, while the yogurt fermented with *L. paracasei* SRX10 received the highest score (8.6). For the overall perception of aroma and for the specific attributes of aroma, no differentiation was observed among the samples and they were all evaluated positively, as their scores ranged between 6.0–8.5 on the hedonic scale. Emphasis was given for the overall perception of taste and texture and their specific sensorial attributes, as these characteristics presented the greatest variability between cases. The results obtained from these two sensorial categories were decisive results for accepting or rejecting a sample. Twenty samples (19 samples and the control) had a pleasant and distinctive taste and were recorded with scores >5 in the hedonic scale for the overall perception of taste, whereas most samples demonstrated better taste compared to the control. These samples were produced by different isolates of *L. lactis* SRX2, SRX3, SRX4, SRX5, SRX17, SMX2, SMX5, and SMX16; *Leuconostoc* sp. SRX8; *L. mesenteroides* FRX4, FRX13, FMX3, FMX6, and SRX9; *L. pseudomesenteroides* SRX1; *L. plantarum* FRX7, FRX20, and FB1; and *L. paracasei* SRX10. Regarding the specific attributes of taste, these samples exhibited a sweet taste and low acidity, with scores similar to the control, and most of them had a slight salty taste, whereas rancidity and bitterness were not detected. Samples that were rejected displayed the high scores in rancidity and bitterness. Most of these rejected samples were assigned to the genus *Leuconostoc* (23 isolates). For the specific characteristics of texture, the samples were examined for their consistency, homogeneity, and grainy texture. Yogurt samples with a bad consistency, as well as with a “foamy texture”, were scored with <5 in the hedonic scale. In total, nine samples were judged as unacceptable, produced by *L. mesenteroides* FRX2, FMX1, and FMX11; *L. pseudomesenteroides* SRX18; *L. plantarum* FRX17 and FB17; *L. lactis* SRX14; and *L. brevis* SRX19 and SRX20. On the other hand, 39 samples were found acceptable, with scores over 5 for the overall perception of texture, whereas nine samples fermented with *L. lactis* SRX2, SRX3, SMX2, SMX5, and SMX16; *L. mesenteroides* SRX6; *Leuconostoc* sp. SRX8; *L. plantarum* FRX20; and *L. paracasei* SRX10 exhibiting the best texture with scores above 7 (Table 2). In total, it was observed that yogurts produced with isolates belonging to *L. mesenteroides* exhibited great variability in the sensory analysis, as some samples were evaluated with high scores in some organoleptic characteristics and others were evaluated as unacceptable, as described above, suggesting intra-species variability.

In general, it is of great importance that the co-cultures/adjunct cultures of autochthonous LAB added in fermented foods (to improve a product’s quality and safety) will not deteriorate the sensory characteristics of the final products. Indigenous LAB can improve the technological properties (EPS and enzyme production, acidification, etc.) of fermented foods, leading to better sensorial characteristics in terms of flavor and texture [65]. According to previous studies, LAB species can play an important role in the development of aroma and taste perception in fermented dairy products, and may produce EPS that can improve the texture, rheology, and firmness (mouth feel) and/or reduce syneresis. These properties of LAB species constitute an advantage to the dairy industry that produce yogurt and cheese [64,65,102,103,104]. Other technological characteristics of LAB that can intensify the texture and aroma of fermented foods is the sugar catabolism, the diacetyl production, and/or the ability of proteolysis and lipolysis [64,65]. Among the sensorial characteristics of yogurts, appearance (color, skin, and syneresis) and color constitute important yogurt parameters, as these are the first characteristics that consumers perceive [105,106]. In the current study, it was observed that isolates that had the ability of EPS production (*L. pseudomesenteroides* SRX1 and *L. lactis* SRX2, SRX3, SRX5, SMX16, and SMX20) and/or were proteolytic (*L. lactis* SRX3, SRX4, SMX2, SMX5, and SXM16), were evaluated with high scores in all the organoleptic characteristics. Accordingly, many studies to date dealing with the addition of probiotic strains as adjunct cultures in yogurts, have studied their effect on specific sensorial characteristics (color, taste, odor and texture) during the storage of the products. For example, Mani-López et al. [106] evaluated six probiotic yogurts in terms of texture, color, and syneresis during cold storage. The results revealed that syneresis was higher at the probiotic yogurts, no color differences were observed between the different probiotic strains used and the control ones, and finally, no texture and flavor differences were identified by the consumers [106]. On the other hand, Saxami et al. [46] used two lactobacilli strains as adjunct cultures in yogurt production, and the results showed that the probiotic products were found to be more acidic, while texture and color were not affected by the probiotic cultures. Nonetheless, in the aforenoted study, the probiotic products were still acceptable from the sensory panel. In addition, Papadopoulou et al. [107] inoculated yogurt with a probiotic *L. plantarum* strain and observed that the probiotic samples were more acidic, especially at the end of the shelf life of the products; however, the texture and odor were similar to the control.

#### 3.8.2. Microbiological and Molecular Analysis

**Figure 2:**
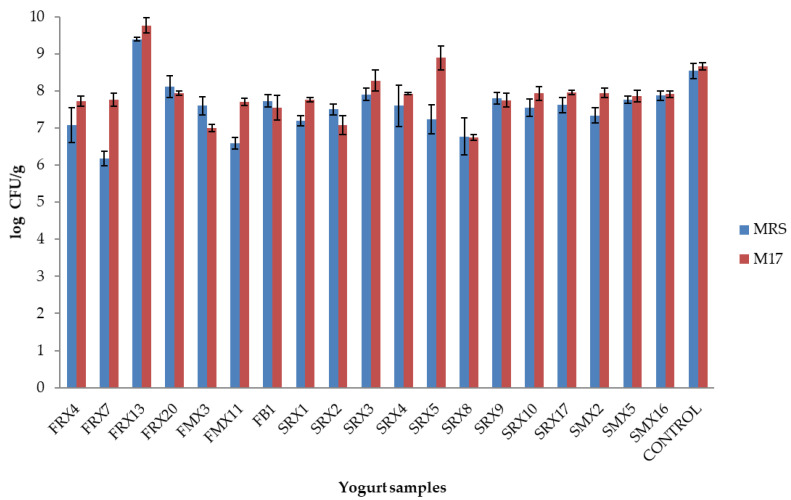
Microbial counts (log CFU/g) of LAB (MRS) and lactic cocci/streptococci (M17) of the control yogurt sample and of the 19 LAB isolates used as co-cultures in the yogurt samples at refrigerated storage, 24 h after production. The error bars represent the standard deviation of the mean values (*n* = 3).

**Figure 3:**
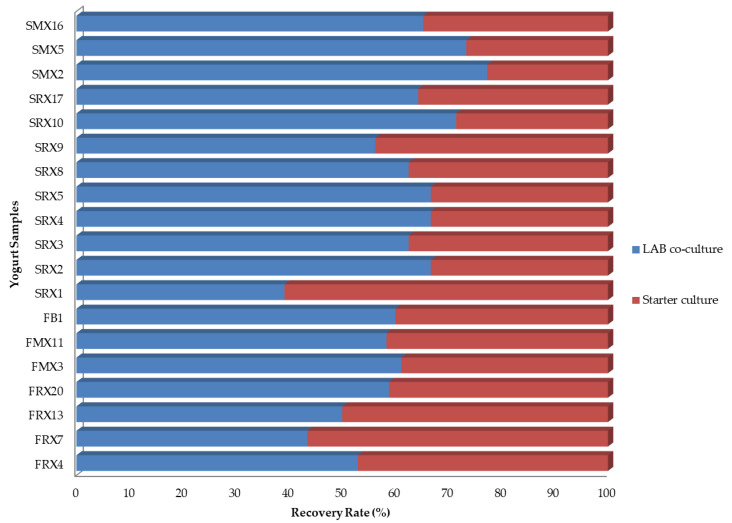
Recovery rates of the 19 LAB isolates, used as co-cultures in yogurt samples after molecular analysis.

The samples with acceptable taste (19 cases and the control case) were subjected to microbiological and molecular analyses. The microbiological analysis (24 h after production) showed that LAB were enumerated in population levels of 6.2–9.5 log CFU/g, depending on the isolate used (Figure 2). For instance, the isolates *L. mesenteroides* FRX13, *L. plantarum* FRX20, and *L. lactis* SRX3 and SRX5 exhibited higher LAB counts (>8.0 log CFU/g), while for yogurts produced with the isolates *L. plantarum* FRX7, *L. mesenteroides* FMX11, and *Leuconostoc* sp. SRX8, LAB populations were detected in lower counts (*ca.* 6.2–6.8 log CFU/g) (Figure 2). The results of the RAPD-PCR fingerprinting revealed that the selected isolates were recovered in all cases in various ratios (39–77%, depending on the case), in comparison to the commercial culture (Figure 3). More specifically, eight isolates of *L. lactis* (SRX2, SRX3, SRX4, SRX5, SRX17, SMX2, SMX5, and SMX16) and one isolate of *L. paracasei* (SRX1) showed a higher recovery rate than the isolates belonging to *L. mesenteroides* (FRX4, FRX13, FMX3, FMX11, SRX8, and SRX9) and *L. plantarum* (FRX7, FRX20, and FB1). On the contrary, *L. pseudomesenteroides* SRX1 exhibited the lowest recovery rate of the 19 samples.

The use of indigenous LAB (isolated from fermented foods) as co-cultures/adjunct cultures/protective cultures for the production of fermented dairy products is reported to be more efficient than the use of commercial cultures, as they have the ability to remain and inhabit their natural environment better and consequently become the dominant microbiota in the products [65]. Moreover, the application of indigenous LAB in dairy product manufacturing can contribute to products with enhanced technological and functional properties (i.e., probiotic properties) [108]. The consumption of probiotic foods offers numerous health benefits to consumers and to achieve their probiotic function to the host, their survival must be ensured during the manufacture and storage of the product with population levels being at least 6 log CFU per g or mL, according to US-FDA [109]. Still, monitoring the survival and the distribution of the added probiotic strains using molecular tools is considered an important aspect to assess the viability of the strains [46,110,111]. To date, studies dealing with various fermented dairy products have evaluated the survival of potential probiotic strains after production and/or during storage. Specifically, Sidira et al. [110] added free and immobilized cells of the probiotic *L. casei* ATCC 393 during yogurt production and used molecular tools (strain-specific multiplex PCR) to monitor the survival of the strains throughout storage. It was evident that both free and immobilized cells of the probiotic strain were viable at high population levels, adequate for conferring health benefits to the consumer, during yogurt storage at 4 °C [110]. Similar results were observed in the study by Saxami et al. [46] after the production of a probiotic yogurt using two probiotic lactobacilli strains (*L. pentosus* B281 and *L. plantarum* B282), where PCR analysis demonstrated that the two probiotic strains were present in yogurt at levels ≥6 log CFU/g until the end of the shelf life of the product.

Nevertheless, the current study was focused on the characterization of a variety of technological and functional properties of the indigenous LAB, as well as on the initial screening of the organoleptic profile of these LAB isolates, when used as co-cultures, using yogurt as a quick fermentation product. In more detail, isolates that presented good survival rates at low pH and bile salts, and the ability to produce *β*-galactosidase and exopolysaccharides (EPS), attributed desirable sensory characteristics to yogurt and displayed great survival rates after fermentation and could be suitable for further studies. Some prominent isolates that exhibited the majority of these features were *L. lactis* (SRX2, SRX3, SRX5, and SMX16), *L. paracasei* SRX10, and *L. plantarum* (FRX7, FB1), while *L. mesenteroides* FMX3 and *L. lactis* SMX2 also presented anti-listerial activity (Table 3). A future goal will be the inclusion of the selected isolates in a larger scale fermentation of novel dairy products.

## 4. Conclusions

The findings of the present study demonstrated that certain indigenous LAB isolates from traditionally produced artisanal dairy products were found to possess desirable properties in vitro and in situ. Many of the 48 selected isolates demonstrated desirable characteristics in vitro, while 19 isolates were evaluated positively in yogurt regarding sensory analysis and also showed great survival rates after the fermentation of the yogurt. Some prominent isolates that showed favorable technological and functional characteristics (good survival rates at low pH and bile salts and had the ability to produce *β*-galactosidase and EPS) and attributed desirable sensory characteristics to yogurt were *L. lactis* (SRX2, SRX3, SRX5, and SMX16), *L. paracasei* SRX10, and *L. plantarum* (FRX7 and FB1), while *L. mesenteroides* FMX3 and *L. lactis* SMX2 showed anti-listerial activity in vitro. It would be of great interest to evaluate the technological and antimicrobial performance of these nine prominent isolates in a real food ecosystem. Future studies are needed to evaluate the applicability of the multi-functional isolates with probiotic potential under different conditions used for the production of novel dairy products with traditional character and distinctive organoleptic properties. In addition, in vivo studies are required to evaluate the survival/attachment of the selected isolates through the gastrointestinal tract of the host, to confirm their probiotic potential. For this direction, clinical trials will be needed to determine the measurable probiotic-induced health benefits.

## Figures and Tables

**Table 1:** Results from the in vitro tests of the 48 selected LAB isolates. MIC values for the selected strains according to the breakpoints set by EFSA (2018) [45].

LAB Isolates	pH 2.5 (SR%) ^1^	Bile Salts (SR%) ^2^	Bile Salts Hydrolysis ^3^	Antimicrobial Activity ^4^	Antibiotic Resistance								
					AM	VM	GM	KM	SM	EM	CL	TC	CM
*L. mesenteroides* FRX1	48%	87%	0	0	≤1	1024	8	64 ^R^	64	≤1	≤1	4	4
*L. mesenteroides* FRX2	53%	65%	0	0	≤1	1024	4	32 ^R^	64	≤1	≤1	4	4
*L. mesenteroides* FRX3	54%	83%	0	0	≤1	1024	4	64 ^R^	32	≤1	≤1	2	4
*L. mesenteroides* FRX4	50%	91%	0	0	≤1	>1024	4	32 ^R^	32	2 ^R^	≤1	4	4
*L. mesenteroides* FRX5	50%	87%	0	0	≤1	1024	4	32 ^R^	32	≤1	≤1	4	4
*L. mesenteroides* FRX6	12%	96%	0	0	≤1	1024	4	64 ^R^	32	≤1	≤1	2	4
*L. plantarum* FRX7	81%	99%	1	0	≤1	>1024	>128 ^R^	>1024 ^R^	>1024	>2 ^R^	2	64 ^R^	>16 ^R^
*L. mesenteroides* FRX8	34%	73%	0	0	≤1	1024	4	32 ^R^	16	≤1	≤1	4	4
*L. mesenteroides* FRX9	23%	91%	0	0	≤1	>1024	4	64 ^R^	64	≤1	≤1	4	4
*L. mesenteroides* FRX10	32%	91%	0	0	≤1	>1024	4	64 ^R^	64	≤1	≤1	4	>16 ^R^
*L. mesenteroides* FRX11	58%	91%	0	0	≤1	>1024	4	64 ^R^	32	≤1	≤1	4	4
*L. mesenteroides* FRX12	50%	75%	0	0	≤1	>1024	4	32 ^R^	32	≤1	≤1	4	4
*L. mesenteroides* FRX13	23%	63%	0	0	≤1	>1024	2	3 ^R^	64	≤1	≤1	2	4
*L. mesenteroides* FRX14	33%	76%	0	0	≤1	1024	2	64 ^R^	128 ^R^	≤1	≤1	2	4
*L. mesenteroides* FRX15	26%	84%	0	0	≤1	>1024	8	64 ^R^	128 ^R^	≤1	≤1	2	4
*L. mesenteroides* FRX16	50%	98%	0	0	≤1	1024	2	32 ^R^	32	≤1	≤1	2	4
*L. plantarum* FRX17	73%	97%	1	0	≤1	>1024	>128 ^R^	>1024 ^R^	512	>2 ^R^	≤1	>128 ^R^	>16 ^R^
*L. mesenteroides* FRX18	53%	93%	0	0	≤1	>1024	4	32 ^R^	32	≤1	≤1	4	4
*L. mesenteroides* FRX19	42%	77%	0	0	≤1	1024	4	32 ^R^	32	≤1	≤1	2	4
*L. plantarum* FRX20	72%	96%	1	0	≤1	>1024	>128 ^R^	>1024 ^R^	256	>2 ^R^	2	>128 ^R^	>16 ^R^
*L. mesenteroides* FMX1	12%	72%	0	0	≤1	1024	4	64 ^R^	64	≤1	≤1	≤1	4
*L. mesenteroides* FMX3	44%	99%	0	1	≤1	1024	128 ^R^	512 ^R^	128 ^R^	>2 ^R^	≤1	≤1	4
*L. mesenteroides* FMX6	54%	76%	0	0	≤1	1024	8	32	128 ^R^	≤1	≤1	≤1	4
*L. mesenteroides* FMX11	51%	79%	0	0	≤1	1024	32 ^R^	64 ^R^	128 ^R^	2 ^R^	≤1	≤1	4
*L. mesenteroides* FMX12	49%	75%	0	0	≤1	>1024	2	32 ^R^	32	≤1	≤1	≤1	4
*L. mesenteroides* FMX14	21%	79%	0	0	≤1	1024	4	64 ^R^	64	≤1	≤1	2	4
*L. plantarum* FB1	55%	96%	1	0	≤1	>1024	>128 ^R^	>1024 ^R^	512	>2 ^R^	≤1	>128 ^R^	>16 ^R^
*L. plantarum* FB17	72%	95%	1	0	≤1	32	>128 ^R^	>1024 ^R^	128	>2 ^R^	2	>128 ^R^	8
*L. pseudomesenteroides* SRX1	55%	98%	0	0	≤1	>1024	64 ^R^	512 ^R^	256 ^R^	2 ^R^	≤1	≤1	4
*L. lactis* SRX2	60%	91%	1	0	≤1	1	8	32	64 ^R^	≤1	≤1	≤1	4
*L. lactis* SRX3	50%	93%	1	0	≤1	1	16	32	64 ^R^	≤1	≤1	≤1	4
*L. lactis* SRX4	48%	100%	1	0	≤1	1	>128 ^R^	>1024 ^R^	256 ^R^	2 ^R^	≤1	≤1	8
*L. lactis* SRX5	54%	97%	1	0	≤1	1	>128 ^R^	>1024 ^R^	256 ^R^	>2 ^R^	≤1	≤1	8
*L. mesenteroides* SRX6	82%	87%	0	0	≤1	1024	4	64 ^R^	64	≤1	≤1	≤1	4
*L. pseudomesenteroides* SRX7	40%	91%	0	0	≤1	1024	64	128	128 ^R^	≤1	≤1	4	4
*Leuconostoc* sp. SRX8	53%	95%	0	0	≤1	1024	4	64 ^R^	128 ^R^	≤1	≤1	≤1	4
*L. mesenteroides* SRX9	43%	92%	0	0	≤1	1024	4	64 ^R^	64	≤1	≤1	4	2
*L. paracasei* SRX10	91%	93%	1	0	≤1	>1024	>128 ^R^	>1024 ^R^	512 ^R^	>2 ^R^	≤1	>128 ^R^	>16 ^R^
*L. lactis* SRX14	64%	99%	0	0	≤1	1	4	128	64 ^R^	≤1	≤1	≤1	4
*L. pseudomesenteroides* SRX16	64%	95%	0	0	≤1	1024	8	64 ^R^	32	≤1	≤1	4	4
*L. lactis* SRX17	51%	90%	0	0	≤1	1	>128 ^R^	>1024 ^R^	256 ^R^	>2 ^R^	≤1	≤1	8
*L. pseudomesenteroides* SRX18	39%	88%	0	0	≤1	>1024	4	64 ^R^	64	≤1	≤1	4	2
*L. brevis* SRX19	67%	88%	1	0	≤1	512	32 ^R^	256 ^R^	128 ^R^	>2 ^R^	≤1	64 ^R^	8 ^R^
*L. brevis* SRX20	51%	91%	1	0	≤1	512	32 ^R^	256 ^R^	128 ^R^	>2 ^R^	≤1	64 ^R^	8 ^R^
*L. lactis* SMX2	45%	99%	1	1	≤1	1	>128 ^R^	>1024 ^R^	256 ^R^	>2 ^R^	≤1	≤1	8
*L. lactis* SMX5	43%	95%	1	0	≤1	1	>128 ^R^	512 ^R^	256 ^R^	>2 ^R^	≤1	≤1	8
*L. lactis* SMX16	54%	95%	1	0	≤1	1	>128 ^R^	>1024 ^R^	256 ^R^	>2 ^R^	≤1	≤1	8
*L. lactis* SMX20	52%	97%	1	0	≤1	1	>128 ^R^	>1024 ^R^	256 ^R^	>2 ^R^	≤1	≤1	8

^1^ SR: survival rate after 3 h in a low pH (2.5); ^2^ SR: survival rate after 4 h in bile salts; ^3^ 0: non hydrolase activity; 1: partial hydrolase activity; ^4^ 0: non antimicrobial activity against *Salmonella* Enteritidis (FMCC-B-56), *Staphylococcus aureus* (FMCC-B-202), and *Listeria monocytogenes* (FMCC-B-129, FMCC-B-131, FMCC-B-133, DSMZ19094, and DSMZ15675); 1: antimicrobial activity against selected *Listeria monocytogenes* strains (FMCC-B-129, FMCC-B-131, FMCC-B-133, DSMZ19094, and DSMZ15675). ^R^: Resistant according to EFSA (2018) breakpoints [45]. AM: ampicillin; VM: vancomycin; GM: gentamicin; KM: kanamycin; SM: streptomycin; EM: erythromycin; CL: clindamycin; TC: tetracycline; CM: chloramphenicol.

**Table 2:** Sensory evaluation in terms of overall perception of the appearance, aroma, taste, and texture of yogurts produced with the commercial culture (control) and with the addition of one of the 48 isolates per case. Values are expressed as mean values ± the standard deviation of the seven-member panel.

LAB Isolates	Overall Appearance	Overall Aroma	Overall Taste	Overall Texture
*L. mesenteroides* FRX1	6.2 ± 0.9	6.6 ± 0.9	4.9 ± 0.1	5.8 ± 0.8
*L. mesenteroides* FRX2	7.5 ± 0.7	6.2 ± 1.0	4.3 ± 0.1	4.8 ± 0.3
*L. mesenteroides* FRX3	5.4 ± 0.8	6.8 ± 0.1	4.2 ± 0.9	6.8 ± 0.1
*L. mesenteroides* FRX4	6.3 ± 0.3	7.3 ± 1.0	5.3 ± 0.3	5.6 ± 0.9
*L. mesenteroides* FRX5	6.7 ± 0.5	5.2 ± 0.2	4.2 ± 0.4	6.8 ± 1.0
*L. mesenteroides* FRX6	6.7 ± 0.8	5.8 ± 0.7	4.6 ± 0.4	6.6 ± 0.9
*L. plantarum* FRX7	5.6 ± 0.6	6.3 ± 0.4	6.5 ± 0.5	5.2 ± 0.3
*L. mesenteroides* FRX8	7.2 ± 0.9	6.2 ± 1.0	4.7 ± 0.4	6.7 ± 0.3
*L. mesenteroides* FRX9	7.0 ± 0.2	6.6 ± 0.5	3.1 ± 0.7	5.6 ± 0.5
*L. mesenteroides* FRX10	5.7 ± 0.6	6.6 ± 0.8	4.3 ± 0.7	5.2 ± 0.2
*L. mesenteroides* FRX11	6.9 ± 1.1	5.2 ± 0.5	4.6 ± 0.5	5.2 ± 0.7
*L. mesenteroides* FRX12	5.1 ± 0.5	6.8 ± 0.8	3.9 ± 0.9	5.0 ± 0.2
*L. mesenteroides* FRX13	6.8 ± 0.3	6.9 ± 0.8	6.7 ± 0.8	6.5 ± 0.5
*L. mesenteroides* FRX14	6.6 ± 1.1	7.1 ± 0.8	4.1 ± 0.6	6.2 ± 0.2
*L. mesenteroides* FRX15	4.9 ± 0.3	7.1 ± 0.4	4.9 ± 0.9	6.7 ± 0.7
*L. mesenteroides* FRX16	6.0 ± 0.2	6.0 ± 0.7	4.1 ± 0.8	5.9 ± 0.8
*L. plantarum* FRX17	3.4 ± 0.6	6.5 ± 0.9	4.8 ± 0.1	4.9 ± 0.9
*L. mesenteroides* FRX18	6.4 ± 0.5	6.2 ± 0.7	4.8 ± 0.3	6.6 ± 0.8
*L. mesenteroides* FRX19	6.5 ± 0.9	7.0 ± 0.8	3.8 ± 0.9	5.7 ± 0.5
*L. plantarum* FRX20	5.1 ± 0.3	7.2 ± 0.4	5.9 ± 0.8	7.4 ± 0.7
*L. mesenteroides* FMX1	4.4 ± 0.9	6.7 ± 0.1	4.5 ± 0.3	4.9 ± 0.7
*L. mesenteroides* FMX3	6.2 ± 1.0	7.2 ± 1.0	6.4 ± 0.9	6.6 ± 0.6
*L. mesenteroides* FMX6	6.9 ± 0.6	7.2 ± 0.8	6.3 ± 0.9	5.8 ± 0.7
*L. mesenteroides* FMX11	4.6 ± 0.3	6.0 ± 0.3	3.6 ± 0.5	3.9 ± 0.4
*L. mesenteroides* FMX12	6.7 ± 0.7	6.4 ± 0.2	3.9 ± 0.2	5.1 ± 0.1
*L. mesenteroides* FMX14	6.5 ± 0.4	5.7 ± 0.8	4.9 ± 0.8	6.2 ± 0.4
*L. plantarum* FB1	7.0 ± 0.2	6.8 ± 0.8	5.5 ± 0.6	5.4 ± 0.5
*L. plantarum* FB17	1.8 ± 0.2	7.2 ± 0.6	3.6 ± 0.3	4.4 ± 0.3
*L. pseudomesenteroides* SRX1	6.2 ± 0.3	7.3 ± 0.8	7.4 ± 0.8	6.7 ± 0.3
*L. lactis* SRX2	5.5 ± 0.1	6.0 ± 0.3	6.7 ± 0.1	7.3 ± 0.5
*L. lactis* SRX3	8.3 ± 0.8	7.1 ± 0.9	7.0 ± 0.9	7.5 ± 0.6
*L. lactis* SRX4	7.4 ± 0.6	8.0 ± 0.9	7.1 ± 0.7	6.9 ± 0.6
*L. lactis* SRX5	8.4 ± 0.3	7.6 ± 0.5	7.2 ± 0.3	6.6 ± 1.0
*L. mesenteroides* SRX6	6.1 ± 0.2	5.0 ± 0.6	4.9 ± 0.7	7.1 ± 0.9
*L. pseudomesenteroides* SRX7	5.4 ± 0.2	6.0 ± 0.2	4.9 ± 0.1	6.5 ± 0.5
*Leuconostoc* sp. SRX8	5.9 ± 0.7	7.9 ± 0.4	8.4 ± 0.8	8.3 ± 0.3
*L. mesenteroides* SRX9	5.5 ± 0.3	5.9 ± 0.9	6.5 ± 0.9	6.1 ± 0.8
*L. paracasei* SRX10	8.6 ± 0.4	8.3 ± 0.7	8.7 ± 0.5	9.0 ± 0.2
*L. lactis* SRX14	3.6 ± 0.4	6.9 ± 0.7	4.2 ± 0.3	2.9 ± 0.6
*L. pseudomesenteroides* SRX16	3.7 ± 0.7	6.8 ± 0.6	3.6 ± 0.6	6.4 ± 0.2
*L. lactis* SRX17	7.0 ± 0.9	7.5 ± 0.9	7.1 ± 0.2	6.6 ± 0.3
*L. pseudomesenteroides* SRX18	6.5 ± 0.6	6.9 ± 0.7	4.2 ± 0.7	4.6 ± 0.9
*L. brevis* SRX19	6.1 ± 0.2	6.6 ± 0.3	3.5 ± 0.8	4.0 ± 0.4
*L. brevis* SRX20	6.0 ± 0.2	6.9 ± 0.6	3.5 ± 0.7	4.2 ± 0.2
*L. lactis* SMX2	5.1 ± 0.6	7.0 ± 0.3	7.7 ± 0.9	7.2 ± 0.7
*L. lactis* SMX5	7.9 ± 0.9	7.7 ± 0.4	7.9 ± 0.7	7.8 ± 0.8
*L. lactis* SMX16	7.3 ± 1.0	7.8 ± 0.9	8.8 ± 0.5	8.6 ± 0.4
*L. lactis* SMX20	5.6 ± 0.4	7.0 ± 0.4	4.0 ± 0.5	4.2 ± 0.4
CONTROL	6.0 ± 0.2	7.4 ± 0.4	5.9 ± 0.1	5.3 ± 0.3

**Table 3:** Results from the in vitro tests and the survival after yogurt fermentation of the final nine selected LAB isolates.

LAB Isolates	pH 2.5 (SR%) ^1^	Bile Salts (SR%) ^2^	Bile Salts Hydrolysis ^3^	Antimicrobial Activity ^4^	Antibiotic Resistance ^5^	*β*-galactosidase ^6^	EPS ^7^	Proteolytic Activity ^8^	Survival after Yogurt Production (RR%) ^9^
*L. plantarum* FRX7	81%	99%	1	0	GM, KM, EM, TC, CM	1	0	1	43%
*L. mesenteroides* FMX3	44%	99%	0	1	GM, KM, SM, EM	1	0	1	61%
*L. plantarum* FB1	55%	96%	1	0	GM, KM, EM, TC, CM	1	0	0	60%
*L. lactis* SRX2	60%	91%	1	0	SM	0	1	0	67%
*L. lactis* SRX3	50%	93%	1	0	SM	0	1	1	63%
*L. lactis* SRX5	54%	97%	1	0	GM, KM, SM, EM	0	1	0	67%
*L. paracasei* SRX10	91%	93%	1	0	GM, KM, SM, EM, TC, CM	0	0	0	71%
*L. lactis* SMX2	45%	99%	1	1	GM, KM, SM, EM	1	0	1	77%
*L. lactis* SMX16	54%	95%	1	0	GM, KM, SM, EM	1	1	1	65%

^1^ SR: survival rate after 3 h in the low pH (2.5); ^2^ SR: survival rate after 4 h in bile salts; ^3^ 0: no hydrolase activity; 1: partial hydrolase activity; ^4^ 0: non antimicrobial activity against *Salmonella* Enteritidis (FMCC-B-56), *Staphylococcus aureus* (FMCC-B-202), and *Listeria monocytogenes* (FMCC-B-129, FMCC-B-131, FMCC-B-133, DSMZ19094, and DSMZ15675); 1: antimicrobial activity against selected *Listeria monocytogenes* strains (FMCC-B-129, FMCC-B-131, FMCC-B-133, DSMZ19094, and DSMZ15675). ^5^ Resistant according to EFSA (2018) breakpoints [45]. AM: ampicillin; VM: vancomycin; GM: gentamicin; KM: kanamycin; SM: streptomycin; EM: erythromycin; CL: clindamycin; TC: tetracycline; CM: chloramphenicol; ^6^ 0: no *β*-galactosidase activity; 1: *β*-galactosidase activity; ^7^ 0: no EPS production; 1: EPS production; ^8^ 0: no proteolytic activity; 1: proteolytic activity; ^9^ RR%: recovery rate after yogurt production using molecular tools.

## Data Availability

Data is contained within the current article and the supplementary material.

## Supplementary Materials

The following supporting information can be downloaded at: https://www.mdpi.com/article/10.3390/microorganisms10020246/s1, Table S1: Biochemical characteristics of the isolates retrieved from fresh white brined cheese. Table S2: Biochemical characteristics of the isolates retrieved from fresh semi hard goat cheese. Figure S1: PCR products of Lactiplantibacillus plantarum FRX7, FRX17, FRX20, FB1 and FB17 isolates obtained from the recA gene assay, and PCR products of *Lacticaseibacillus paracasei* SRX10 isolate obtained from *tuf* gene assay.
