# Supplementary material for: Mapping the Key Technological and Functional Characteristics of Indigenous Lactic Acid Bacteria Isolated from Greek Traditional Dairy Products
Source: Microorganisms. 2022 Jan 23;10(2):246. doi: 10.3390/microorganisms10020246 (PMC8875946; doi:10.3390/microorganisms10020246)
Supplement: Supplementary file 1 [file microorganisms-10-00246-s001.zip › microorganisms-1501272-supplementary.pdf]

Supplementary Tables

Table S1. Biochemical characteristics of the isolates retrieved from fresh white brined cheese.

Isolates		Isolated medium/ Temperature °C	10 °C	42 °C	pH 4.4	pH 9.6	Anaerobic test	CO_2	6.5% NaCl	Haemolytic activity	Diacetyl Production	Lipolytic activity	Proteolytic Activity	EPS production
FRX1	*Leuconostoc mesenteroides*	MRS/ 30 °C	+	-	+	-	+	+	-	γ	-	-	+	-
FRX2	*Leuconostoc mesenteroides*		+	-	+	-	+	+	+	γ	-	-	+	-
FRX3	*Leuconostoc mesenteroides*		+	-	+	-	+	+	+	γ	-	-	+	-
FRX4	*Leuconostoc mesenteroides*		+	-	+	-	+	+	+	γ	-	-	+	-
FRX5	*Leuconostoc mesenteroides*		+	-	+	-	+	+	+	γ	-	-	+	-
FRX6	*Leuconostoc mesenteroides*		+	-	+	-	+	+	-	γ	-	-	+	-
FRX7	*Lactiplantibacillus plantarum*		+	-	+	-	+	-	+	γ	-	-	+	-
FRX8	*Leuconostoc mesenteroides*		+	-	+	-	+	+	+	γ	-	-	+	-
FRX9	*Leuconostoc mesenteroides*		+	-	+	-	+	+	+	γ	-	-	+	-
FRX10	*Leuconostoc mesenteroides*		+	-	+	-	+	+	+	γ	-	-	+	-
FRX11	*Leuconostoc mesenteroides*		+	-	+	-	+	+	+	γ	-	-	+	-
FRX12	*Leuconostoc mesenteroides*		+	-	+	-	+	+	+	γ	-	-	-	-
FRX13	*Leuconostoc mesenteroides*		+	-	-	-	+	+	+	γ	-	-	-	-
FRX14	*Leuconostoc mesenteroides*		+	-	-	-	+	+	-	γ	-	-	-	-
FRX15	*Leuconostoc mesenteroides*		+	-	+	-	+	+	+	γ	-	-	-	-
FRX16	*Leuconostoc mesenteroides*		+	-	+	-	+	+	+	γ	-	-	-	-
FRX17	*Lactiplantibacillus plantarum*		+	-	+	-	+	-	+	γ	-	-	-	-
FRX18	*Leuconostoc mesenteroides*		+	-	+	-	+	-	+	γ	-	-	-	-
FRX19	*Leuconostoc mesenteroides*		+	-	+	-	+	+	-	γ	-	-	-	-
FRX20	*Lactiplantibacillus plantarum*		+	-	+	-	+	-	+	γ	-	-	-	-
FRM1	putative enterococci	MRS/ 42 °C	+	+	+	+	+	-	+	γ	-	-	-	+
FRM4	putative enterococci		+	+	+	+	+	-	+	γ	-	-	-	+
FRM5	putative enterococci		+	+	+	+	+	-	+	γ	-	-	+	+

FRM7	putative enterococci		+	+	+	+	+	-	+	γ	-	-	-	-
FRM15	putative enterococci		+	+	+	+	+	-	+	-	-	-	-	+
FMX1	*Leuconostoc mesenteroides*	M17/ 37 °C	+	-	-	-	+	+	-	γ	-	-	+	-
FMX2	putative enterococci		+	+	+	+	+	+	+	-	-	-	+	-
FMX3	*Leuconostoc mesenteroides*		+	+	+	-	+	-	+	γ	-	-	+	-
FMX4	putative enterococci		+	+	+	+	+	-	+	γ	-	-	+	-
FMX5	putative enterococci		+	+	+	+	+	+	+	γ	-	-	-	-
FMX6	*Leuconostoc mesenteroides*		+	-	+	-	+	+	-	-	-	-	+	-
FMX8	putative enterococci		+	+	+	+	+	+	+	γ	-	-	-	-
FMX9	putative enterococci		+	+	+	+	+	-	+	-	-	-	-	-
FMX10	putative enterococci		+	+	+	+	+	+	+	γ	-	-	-	-
FMX11	*Leuconostoc mesenteroides*		+	-	+	-	+	+	-	-	-	-	-	-
FMX12	*Leuconostoc mesenteroides*		+	+	+	-	+	+	-	γ	-	-	+	-
FMX14	*Leuconostoc mesenteroides*		+	-	+	-	+	-	-	γ	-	-	+	-
FMM2	putative enterococci	M17/ 42 °C	+	+	-	+	+	-	+	γ	-	-	-	-
FMM3	*Enterococcus faecium*		+	+	-	+	+	-	+	γ	-	-	+	-
FMM4	putative enterococci		+	+	-	+	+	-	+	γ	-	-	-	-
FMM5	putative enterococci		+	+	-	+	+	-	+	γ	-	-	+	-
FMM6	putative enterococci		+	+	-	+	+	-	+	γ	-	-	+	-
FMM7	putative enterococci		+	+	-	+	+	-	+	γ	-	-	-	-
FMM8	putative enterococci		+	+	-	+	+	-	+	γ	-	-	+	-
FMM9	putative enterococci		+	+	-	+	+	-	+	γ	-	-	-	-
FMM10	putative enterococci		+	+	-	+	+	-	+	γ	-	-	+	+
FMM12	putative enterococci		+	+	-	+	+	-	+	γ	-	-	+	+
FMM13	putative enterococci		+	+	-	+	+	-	+	γ	-	-	+	-
FMM15	putative enterococci		+	+	-	+	+	-	+	γ	-	-	+	+
FB1	*Lactiplantibacillus plantarum*	M17/ 37 °C	+	+	+	-	+	-	+	γ	-	-	-	-
FB2	*Enterococcus* spp.		+	+	+	+	+	-	+	γ	-	-	+	+
FB3	putative enterococci		+	+	+	+	+	-	+	γ	-	-	+	-
FB4	putative enterococci		+	+	+	+	+	-	+	γ	-	-	-	-

FB5	putative enterococci		+	+	+	+	+	-	+	γ	-	-	-	-
FB6	putative enterococci		+	+	+	+	+	-	+	γ	-	-	+	-
FB7	putative enterococci		+	+	-	+	+	-	+	γ	-	-	+	-
FB9	putative enterococci		+	+	+	+	+	-	+	γ	-	-	+	-
FB10	putative enterococci		+	+	+	+	+	-	+	γ	-	-	+	-
FB11	*Enterococcus faecium*		+	+	+	+	+	-	+	γ	-	-	+	-
FB12	putative enterococci		+	+	+	+	+	-	+	γ	-	-	+	-
FB13	putative enterococci		+	+	+	+	+	-	+	γ	-	-	-	-
FB14	putative enterococci		+	+	+	+	+	-	+	γ	-	-	-	-
FB15	*Enterococcus faecium*		+	+	+	+	+	-	+	γ	-	-	+	-
FB17	*Lactiplantibacillus plantarum*		+	+	+	-	+	-	+	γ	-	-	+	-

Table S2. Biochemical characteristics of the isolates retrieved from fresh semi hard goat cheese.

Isolates		Isolated medium/ Temperature °C	10 °C	42 °C	pH 4.4	pH 9.6	Anaerobic test	CO_2	6.5% NaCl	Haemolytic activity	Diacetyl Production	Lipolytic activity	Proteolytic Activity	EPS production
SRX1	*Leuconostoc pseudomesenteroides*		+	-	+	-	+	+	-	γ	-	-	-	+
SRX2	*Lactococcus lactis*		+	-	+	-	+	-	-	-	-	-	-	+
SRX3	*Lactococcus lactis*		+	-	+	-	+	-	-	-	-	-	+	+
SRX4	*Lactococcus lactis*		+	-	-	-	+	-	-	γ	-	-	+	-
SRX5	*Lactococcus lactis*		+	-	-	-	+	-	-	-	-	-	-	+
SRX6	*Leuconostoc mesenteroides*	MRS/ 30 °C	+	-	+	-	+	+	-	γ	-	-	-	-
SRX7	*Leuconostoc pseudomesenteroides*		+	-	+	-	+	+	-	γ	-	-	-	-
SRX8	*Leuconostoc* spp.		+	-	+	-	+	+	-	γ	-	-	-	-
SRX9	*Leuconostoc mesenteroides*		+	-	+	-	+	-	+	γ	-	-	-	-
SRX10	*Lacticaseibacillus paracasei*		+	-	+	-	+	-	-	γ	-	-	-	-
SRX11	putative enterococci		+	+	-	+	+	-	+	γ	-	-	-	-

SRX12	putative enterococci		+	+	-	+	+	-	+	-	-	-	-	-
SRX13	putative enterococci		+	+	-	+	+	-	+	γ	-	-	-	-
SRX14	*Lactococcus lactis*		+	-	-	-	+	-	-	-	-	-	-	-
SRX16	*Leuconostoc pseudomesenteroides*		+	-	-	-	+	+	-	γ	-	-	-	-
SRX17	*Lactococcus lactis*		+	-	-	-	+	-	-	γ	-	-	-	-
SRX18	*Leuconostoc pseudomesenteroides*		+	-	+	-	+	+	-	γ	-	-	-	-
SRX19	*Levilactobacillus brevis*		+	+	+	-	+	+	-	γ	-	-	-	-
SRX20	*Levilactobacillus brevis*		+	+	+	-	+	+	-	γ	-	-	-	-
SMX2	*Lactococcus lactis*	M17/ 37 °C	+	+	+	-	+	-	-	γ	-	-	+	-
SMX3	putative enterococci		+	+	+	+	+	-	+	γ	-	-	+	-
SMX4	*Enterococcus faecium*		+	+	+	+	+	-	+	γ	-	-	+	-
SMX5	*Lactococcus lactis*		+	+	+	-	+	-	-	γ	-	-	+	-
SMX6	putative enterococci		+	+	+	+	+	-	+	γ	-	-	+	-
SMX8	putative enterococci		+	+	+	+	+	-	+	γ	-	-	+	-
SMX13	putative enterococci		+	+	+	+	+	-	+	γ	-	-	+	-
SMX14	putative enterococci		+	+	+	+	+	-	+	γ	-	-	+	-
SMX15	putative enterococci		+	+	+	+	+	-	+	γ	-	-	+	-
SMX16	*Lactococcus lactis*		+	+	+	-	+	-	-	γ	-	-	+	+
SMX17	putative enterococci		+	+	+	+	+	-	+	γ	-	-	+	+
SMX18	putative enterococci		+	+	+	+	+	-	+	γ	-	-	+	+
SMX19	putative enterococci		+	+	+	+	+	-	+	γ	-	-	+	+
SMX20	*Lactococcus lactis*		+	+	+	-	+	-	-	γ	-	-	+	+
SRM1	putative enterococci	MRS/ 42 °C	+	+	+	+	+	-	+	γ	-	-	+	+
SRM3	putative enterococci		+	+	+	+	+	-	+	γ	-	-	+	+
SB1	putative enterococci	M17/ 37 °C	+	+	+	+	+	-	+	γ	-	-	+	-
SB2	putative enterococci		+	+	+	+	+	-	+	γ	-	-	+	-
SB3	putative enterococci		+	+	+	+	+	-	+	γ	-	-	+	-
SB4	putative enterococci		+	+	+	+	+	-	+	-	-	-	+	-
SB5	putative enterococci		+	+	+	+	+	-	+	γ	-	-	+	-

SB6	putative enterococci		+	+	+	+	+	-	+	γ	-	-	+	-
SB7	putative enterococci		+	+	+	+	+	-	+	γ	-	-	+	-
SB8	*Enterococcus lactis*		+	+	+	+	+	-	+	γ	-	-	+	-
SB9	putative enterococci		+	+	+	+	+	-	+	γ	-	-	+	-
SB10	putative enterococci		+	+	+	+	+	-	+	γ	-	-	+	-

Supplementary Figure

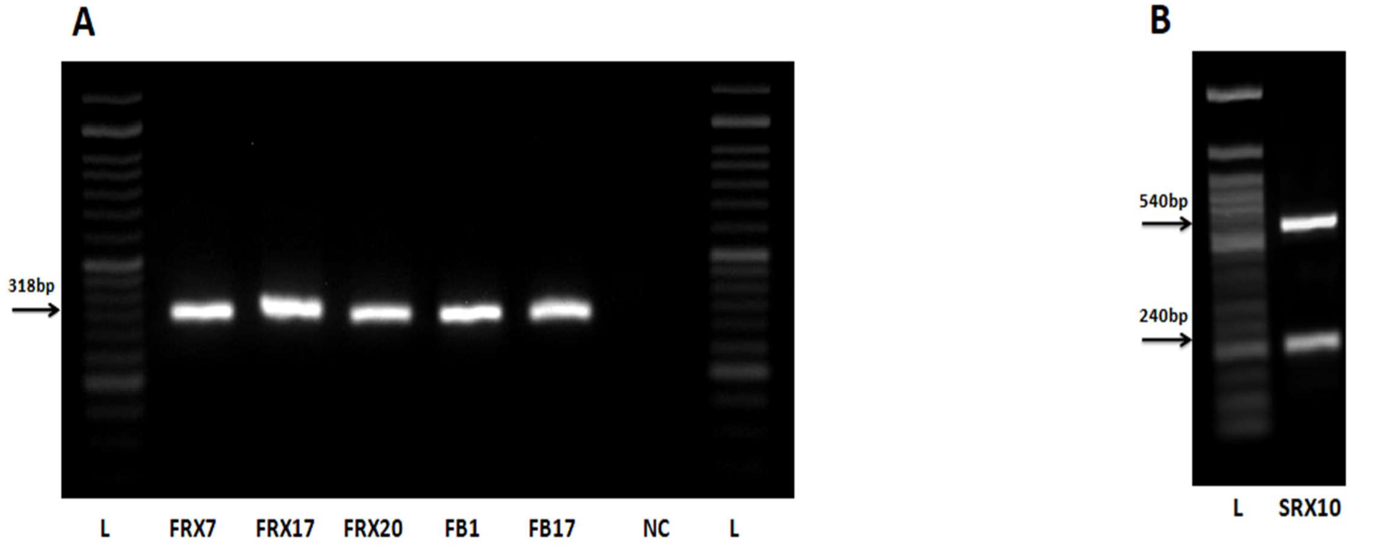

Figure S1. (**A**) PCR products of *Lactiplantibacillus plantarum* FRX7, FRX17, FRX20, FB1 and FB17 isolates obtained from the *rec*A gene assay. The arrow indicates the band of 318bp. L: 50bp DNA ladder (New England, BioLabs); NC: negative control. (**B**) PCR products of *Lacticaseibacillus paracasei* SRX10 isolate obtained from *tuf* gene assay. The arrows indicate the band of 540bp and 240bp. L: 50bp DNA ladder (New England, BioLabs)
